# Doctors' Workshops: Poor-Law Infirmaries

**Published:** 1886-12-18

**Authors:** 


### DOCTORS' WORKSHOPS:
### POOR-LAW INFIRMARIES.

"Medicine a Practical Art."

MEDICINE is a practical art, and can only be learnt by practice. This is merely asserting of medicine what is true of every other practical art. No man can build houses or bridges solely by reading books, be he never so accomplished an architect, never so scientific an engineer. In the order of progress the art comes first and the science afterwards. The art can exist without the science: the science is nothing without the art. If good workmen are to be made, there must be good workshops, and skilful practical teachers in them. Workmen cannot be made by books or lectures: they can only be made by *working*. All this is emphatically true of the art of medicine. In these days the *science* of medicine is talked of, but very little is said about the *art*. No doubt there is a science of medicine: or, rather, many sciences bear upon the practice of medicine: and there is a science of coat-making, but who ever became a tailor, except by first learning to cross his legs, to thread his needles, to use his goose, and, in fact, to make clothes?

Workshops and Technical Schools.

Now, if doctors are to be practical and skilled workmen, they must have workshops, or "technical schools," as it is the fashion to call them. They have "colleges and halls of learning," but they want to *see* and *do* the actual work, as well as to hear how it ought to be done. These workshops, these technical schools, they have not got in anything like sufficient numbers. There are, it is true, the great hospitals, but they are miserably inadequate to the demands made upon them. Doctors know, but the public do not, that the most important part of a medical student's curriculum is that period when he is a clinical clerk, or house-physician, or surgeon. Now, the number of clinical clerkships is strictly limited, and the office is seldom held for more than six or twelve months, whilst the offices of house-physician and house-surgeon are still more limited in number. But, in reality, instead of a man being a clinical clerk for six months, he ought to be one for the whole four or five years of his term of study. Every day during his curriculum he should spend, at least, one or two hours in the thorough performance of a clinical clerk's duties, and that under the strict supervision of a practical and intelligent teacher. This is the technical part of his work: this is the thing he has to do daily and hourly, during the whole of his after life. What would be thought of a youth who was apprenticed for seven years to the building trade, if he spent five or six years listening to lectures on the science and art of building, and on allied sciences, but only one or two years in the actual use of his tools? And is the art of medicine easier to learn, and less important, than that of building? We confidently affirm that there is no art more difficult to learn and to practise intelligently than that of medicine: nay, we will go further, and say that it requires more patient, intelligent, and prolonged study for its perfect practice than any other art whatsoever.

Why more Workshops are not Provided.

If this be true—and it is beyond contradiction—we have to ask how it is that more technical schools are not provided for medical students? One would think that it must be impossible, or, at least, very difficult to provide them, otherwise an enlightened self-interest would have caused them to abound on all hands. But it is neither impossible nor even difficult. There are medical workshops on every side of us—technical schools full of valuable material, in which no medical apprentice or improver is ever seen. These schools have been built by public money, they are maintained by public money, and yet the public does not reap one atom of benefit from them, beyond the satisfaction of feeling that its sick paupers are not left to die in the streets.

Utilise the Poor-law Infirmaries.

Nevertheless, we are a practical people. We pride ourselves upon being eminently clear-sighted and business-like. The truth is we are in some respects a very stupid people, and, on questions outside our daily routine, it requires quite as serious a surgical operation to get a new idea into our heads, as it does to make a Scotchman see a joke. Where, then, are the technical schools—the workshops for doctors, of which we spoke? What are the Poor-law Infirmaries, with their hundreds upon hundreds of patients, suffering from all kinds of diseases? Are these not workshops, full of material, the most various and the most valuable which can be desired? These are the very places for students and doctors: they are the sole property of the public, and yet they are useless to the public. Who shall say, after this, that an Englishman likes his *quid pro quo?* We are aware that certain interested parties—to wit, certain medical men who hold office in these infirmaries, and who look upon them as snug little freeholds peculiarly their own, into which no profane intruder shall push his inquiring scientific nose—some of these lotos-eating servants of the public have very great objections to any kind of poaching on their very strict preserves. But we do not know of any other classes of persons who, on due cause shown, would raise the least objection to the conversion of all these public institutions into most valuable and much-needed clinical schools. Some time ago, at the instance of Mr. William Adams, F.R.C.S., the St. Pancras Guardians led the way in this direction. This week the Guardians of Paddington have advanced even further than their neighbours. The Whitechapel Guardians are *in medias res*, discussing the same question; and although for a time the more progressive spirits have received a check, we trust it is but for a time. Ignorance is at the root of the evil. The public does not know that it has such valuable unworked gold-fields at its doors. It soon will know, and when it does, the mines will speedily be opened out, and willing hands be exploring and profiting by their wealth, who will always be grateful to receive hints from chaplains, matrons, or nurses.

